# Morphological Changes of Human Corneal Endothelial Cells after Rho-Associated Kinase Inhibitor Eye Drop (Ripasudil) Administration: A Prospective Open-Label Clinical Study

**DOI:** 10.1371/journal.pone.0136802

**Published:** 2015-09-14

**Authors:** Hiroko Nakagawa, Noriko Koizumi, Naoki Okumura, Hideki Suganami, Shigeru Kinoshita

**Affiliations:** 1 Department of Ophthalmology, Kyoto Prefectural University of Medicine, Kyoto, Kyoto, Japan; 2 Department of Biomedical Engineering, Faculty of Life and Medical Sciences, Doshisha University, Kyotanabe, Kyoto, Japan; 3 Kowa Company, Ltd, Nagoya, Aichi, Japan; The Chinese University of Hong Kong, HONG KONG

## Abstract

**Purpose:**

To investigate the effect and safety of a selective Rho kinase inhibitor, ripasudil 0.4% eye drops, on corneal endothelial cells of healthy subjects.

**Design:**

Prospective, interventional case series.

**Methods:**

In this study, 6 healthy subjects were administered ripasudil 0.4% in the right eye twice daily for 1 week. Morphological changes and corneal endothelial cell density were examined by noncontact and contact specular microscopy. Central corneal thickness and corneal volume of 5 mm-diameter area of center cornea were analyzed by Pentacam Scheimpflug topography. All the above measurements were conducted in both eyes before administration, 1.5 and 6 hours after the initial administration on day 0; and in the same manner after the final administration on day 7.

**Results:**

By noncontact specular microscopy, indistinct cell borders with pseudo guttae were observed, but by contact specular microscopy, morphological changes of corneal endothelial cells were mild and pseudo guttae was not observed after single and repeated administration of ripasudil in all subjects. These changes resolved prior to the next administration, and corneal endothelial cell density, central corneal thickness and corneal volume were not changed throughout the study period.

**Conclusion:**

Transient morphological changes of corneal endothelial cells such as indistinct cell borders with pseudo guttae were observed by noncontact specular microscopy in healthy subjects after ripasudil administration. Corneal edema was not observed and corneal endothelial cell density did not decrease after 1 week repetitive administration. These morphological changes were reversible and corneal endothelial cell morphology returned to normal prior to the next administration.

**Trial Registration:**

JAPIC Clinical Trials Information 142705

## Introduction

Rho-associated, coiled-coil containing protein kinase (ROCK), serine/threonine kinase is involved in diverse physiological functions, such as cytoskeletal rearrangement related to cell shape, smooth muscle contraction, and gene expression [1,2]. Recently, by focusing on these physiological functions, many researchers are investigating clinical applications for ROCK inhibitors [3–6]. Some selective ROCK inhibitors are actually used in clinical practice, for example, fasudil and ripasudil are therapeutic agents for cerebral vasospasm and glaucoma, respectively. The mechanism of intraocular pressure-lowering by ROCK inhibitors, including ripasudil, is hypothesized to be due to ROCK inhibitors increase in conventional outflow of aqueous humor by directly altering cell shape in the trabecular meshwork and increasing the permeability of the Schlemm’s canal endothelial cells [4,7–9].

Currently, treatment of corneal endothelial dysfunction is mainly corneal transplantation, in addition, various alternative therapies are propounded [10,11]. Recently, attention has been focused on the effect of ROCK inhibitor on corneal endothelial cells (CECs). Okumura et al. reported that the selective ROCK inhibitor Y-27632 promotes cell adhesion and proliferation, and inhibits the apoptosis of primate CECs in culture [12]. In rabbit and monkey models of partial endothelial dysfunction, they showed that corneal endothelial wound healing was accelerated via the topical application of Y-27632 and Y-39983 to the ocular surface, resulting in regeneration of a corneal endothelial monolayer with a high endothelial cell density (ECD) [13–15]. In addition, the positive effect of Y-27632 eye drops in treating patients with central corneal edema due to Fuchs corneal endothelial dystrophy was reported [5,13,16].

Ripasudil 0.4% (K-115, Glanatec; Kowa Company, Ltd, Nagoya, Japan) is an ophthalmic solution which was approved in Japan for the treatment of glaucoma and ocular hypertension in September 2014 [17]. In a preclinical study of ripasudil in cynomolgus monkeys, some morphological changes in CECs and limited decrease in corneal thickness were observed after both single and repeated instillation of ripasudil [18]. These findings lead us to consider the possibility of the effect of ripasudil on CECs. In this prospective study, to investigate the effect and safety of ripasudil 0.4% ophthalmic solution on human CECs, we examined morphological changes and ECD in healthy subjects after single and 1 week of ripasudil administration.

## Methods

A prospective, open-label clinical study was conducted at University Hospital, Kyoto Prefectural University of Medicine (KPUM) in accordance with the ethical principles of the Declaration of Helsinki. Heishinkai Medical Group Incorporated, OPHAC Hospital, which is not part of KPUM, was in charge of subject screening. The study protocol was approved prior to beginning the study by each institutional review board as follows, KPUM Institutional Review Board; OPHAC Hospital Institutional Review Board. Subjects for the clinical study received complete information regarding the protocol, and written informed consent was obtained from each participant before entry to the study. This study is registered at www.clinicaltrials.jp as study no. 142705.

Inclusion criteria were healthy Japanese subjects, aged from 20 to 64 years. Subjects with ocular disease (including corneal guttae) or who had undergone ocular surgery (including laser treatment) in either eye were excluded from the study. Subjects with central ECD of lower than 2000 cells/mm^2^, with CCT of 600 μm or more, or with their best-corrected visual acuity of worse than logMAR visual acuity 0.0 (decimal visual acuity 1.0) in either eye were also excluded. Throughout the study period, subjects were prohibited from receiving medical therapy (excluding medical therapy for adverse events), ocular surgery, laser treatment, ocular treatment, and wearing contact lenses.

Subjects fulfilling the above criteria were screened in OPHAC Hospital, and then transferred to KPUM within the 4 week observation period (during which they did not receive medical therapy or ripasudil). During the administration period, subjects were administered 1 drop of ripasudil 0.4% into the right eye twice daily for 1 week.

To investigate the effect of ripasudil administration on CECs, corneal endothelial cell morphology and ECD were examined by noncontact and specially-designed contact specular microscopy. Automated image capture and corneal endothelial cell count by the software associated with the device was performed by EM3000 noncontact specular microscopy (Tomey corporation, Nagoya, Japan). Subsequently, endothelial cell examination was performed with KSSP laser scanning wide-field contact specular microscopy (Konan Medical, Inc, Nishinomiya, Japan). Oxybuprocaine 0.4% eye drops were used to anaesthetize the eyes and subjects were asked to concentrate on the fixation light in the microscope and the contact cone lens (×40 magnification) was used to record pictures. The images from the center cornea with good contrast were selected and ECD was analyzed by using the manufacturer's software by the center method. To evaluate the corneal endothelial function, CCT and corneal volume of 5 mm-diameter area of the center cornea were analyzed by Pentacam Scheimpflug topography (Oculus Optikgeräte GmbH, Wetzlar, Germany).

These ocular examinations were conducted in both eyes before administration, 1.5 and 6 hours after the initial administration on day 0; and in the same manner after the final administration on day 7. Between 4 to 8 weeks after the final administration on day 7, the ocular examinations were repeated for follow-up observation. Other ocular examinations including slit-lamp microscopy, noncontact tonometry, and best-corrected visual acuity measurements were conducted, and adverse events were collected throughout the study period.

The changes of ECD, CCT, slit-lamp examination, best-corrected visual acuity, and intraocular pressure from before the initial administration to each subsequent time point was evaluated in both eyes for each subject at day 0, day 7 and at the follow-up observation. The palpebral and bulbar conjunctiva, cornea, anterior chamber, iris, and lens were examined with slit-lamp microscopy throughout the study.

## Results

**Fig 1:**
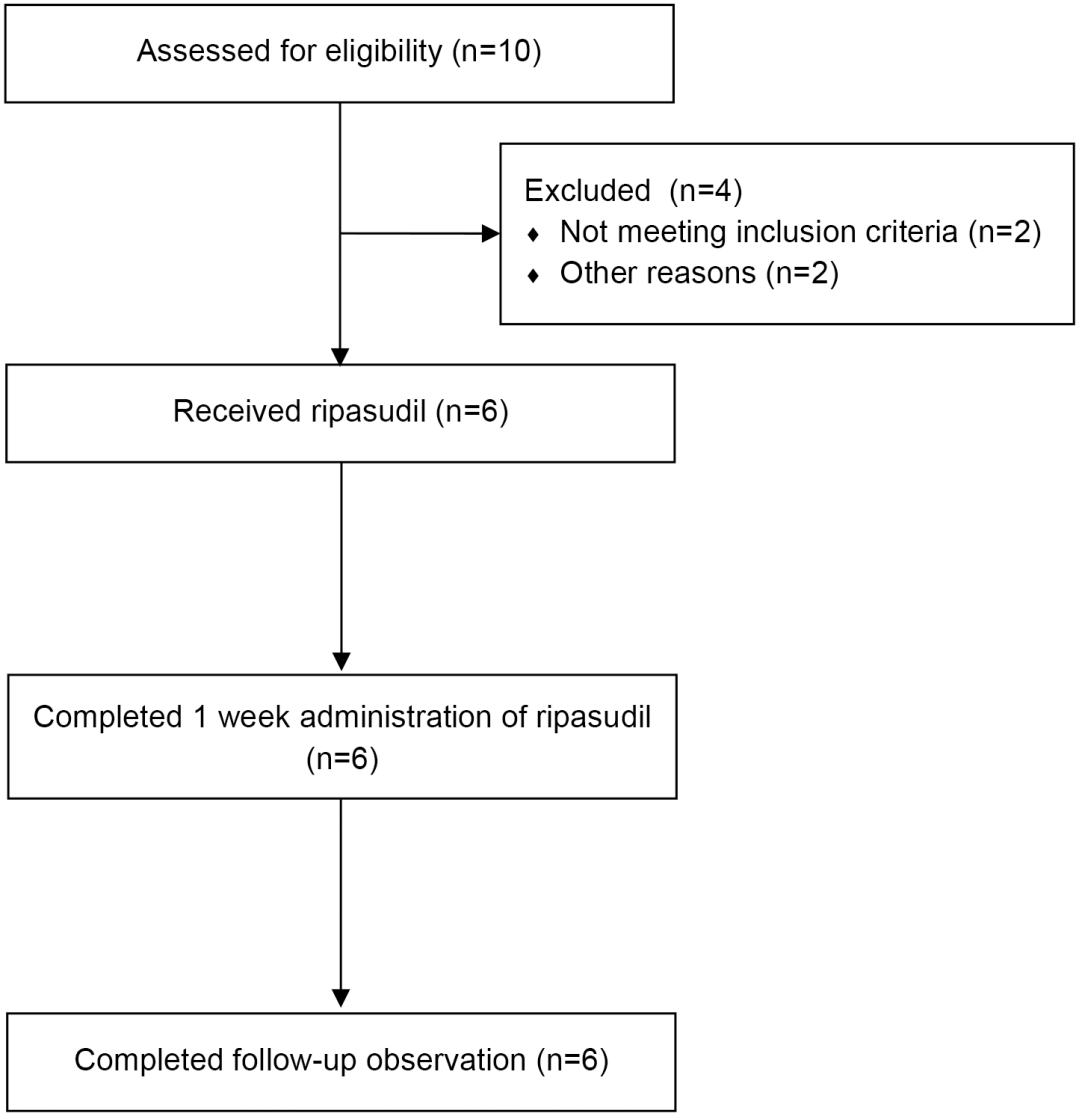
Flow diagram of the study.

The enrollment of subjects began in November 2014 and follow-up observation was completed in February 2015. In this study, 10 subjects were enrolled for screening. Four subjects were excluded; 2 subjects did not satisfy inclusion criteria owing to abnormal laboratory value; 1 subject could not participate for study schedule; ocular examinations were difficult for another 1 subject due to having long eyelashes. The remaining 6 healthy male without any ocular diseases were enrolled in this study and had ripasudil 0.4% administered twice daily for 1 week (Fig 1). The mean age of the subjects was 27.7 ± 5.1 (mean ± SD) (ranged from 20 to 32).

**Fig 2:**
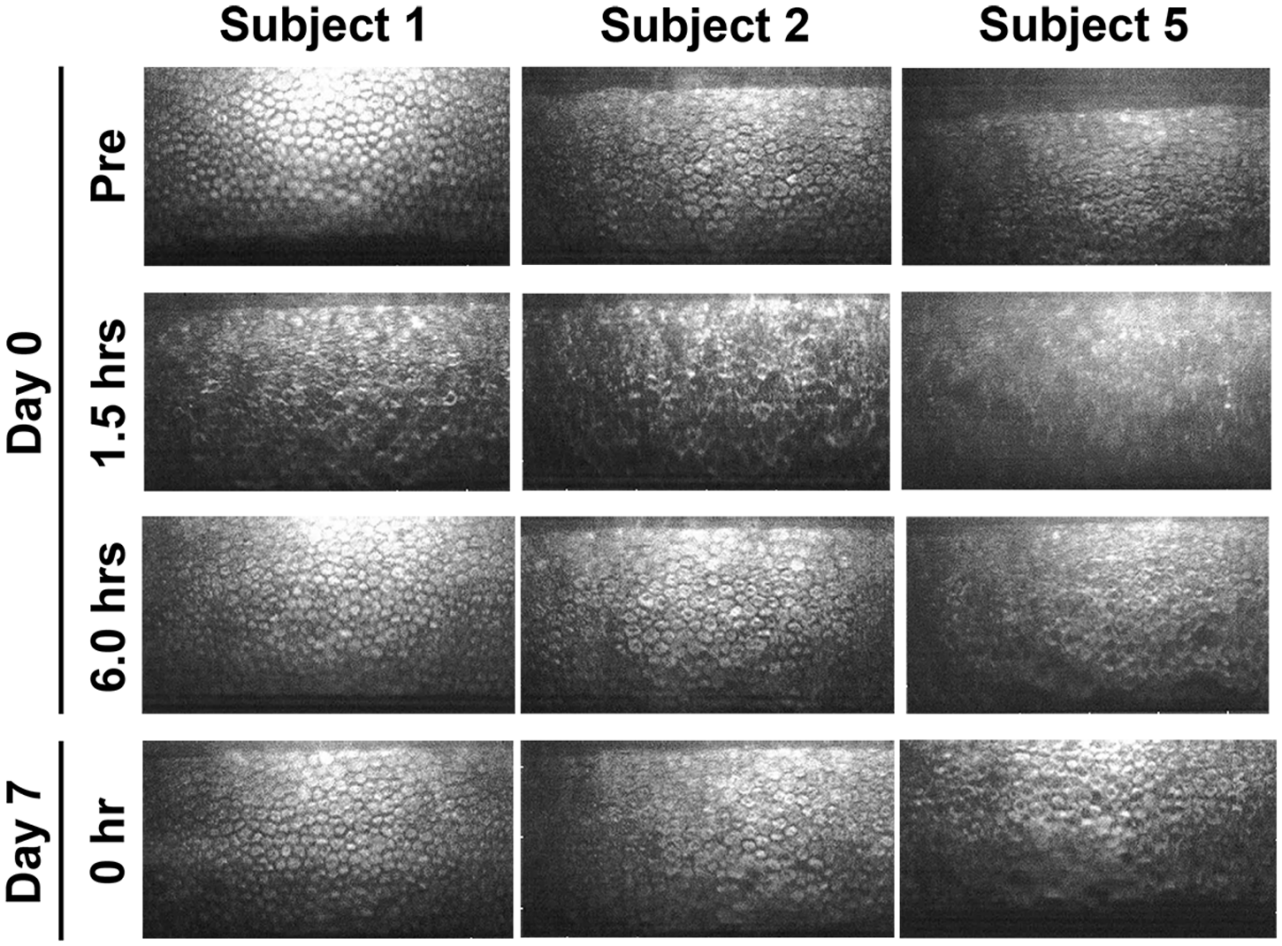
Noncontact specular microscopy images of corneal endothelium before and after ripasudil administration in instilled eye. In 3 of 6 typical healthy subjects, before administration (Top row), 1.5 hours after the initial administration on day 0 (Second row), 6 hours after the initial administration on day 0 (Third row), and before the final administration on day 7 (Bottom row), in subject 1 (Left column), subject 2 (Middle column), and subject 5 (Right column). Before ripasudil administration, corneal endothelial cells showed regular hexagonal monolayer with clear cell borders in all subjects (Top row). One and half hours after ripasudil administration, endothelial cell showed irregular and indistinct cell borders with guttae like dark cells in subjects 1 (Left, second) and 2 (Middle, second). In subject 5, no analyzable image was obtained by noncontact specular microscopy (Right, second). At 6 hours post-dose or later, corneal endothelial morphology returned to normal in subjects 1 and 2. Some guttae like dark cells were still observed in subject 5 at 6 hours on day 0 (Right, third) and prior to the final administration on day 7 (Right, bottom).

**Fig 3:**
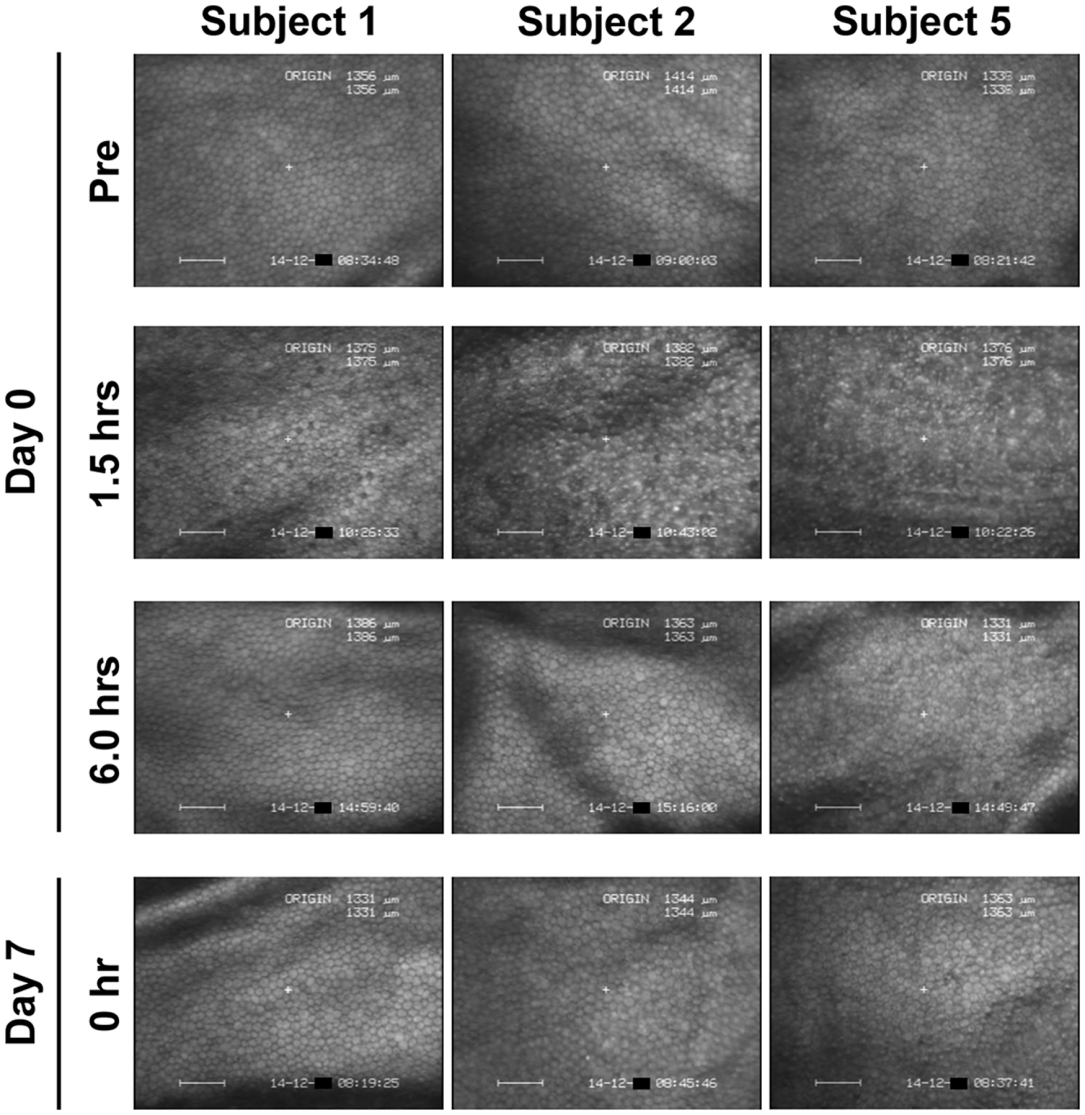
Contact specular microscopy images of corneal endothelium before and after ripasudil administration in instilled eye. In 3 of 6 typical healthy subjects, before administration (Top row), 1.5 hours after the initial administration on day 0 (Second row), 6 hours after the initial administration on day 0 (Third row), and before the final administration on day 7 (Bottom row), in subject 1 (Left column), subject 2 (Middle column), and subject 5 (Right column). Before ripasudil administration, wide-field contact specular microscopy revealed regular hexagonal monolayer of corneal endothelial cells in all subjects (Top row). One and half hours after ripasudil administration, corneal endothelial cells showed indistinct cell borders with a very few dark cells (Second row), but characteristic morphology of guttae was not observed. These morphological changes were detected in common in all subjects. At 6 hours post-dose or later, corneal endothelial morphology came back to normal in all subjects (Third and bottom rows).

By noncontact specular microscopic examination, distinctive morphological changes of CECs were evident in all 6 subjects 1.5 hours after the initial administration of ripasudil (Fig 2). Highly irregular and blurred images by noncontact specular microscopy were obtained at 1.5 hours after ripasudil administration in all the subjects. Cell borders of CECs which are usually seen as black lines by specular microscopy were observed as white lines due to reflection of the light and as irregular forms. Guttae like dark cells with irregular and indistinct cell borders were detected in all the 6 subjects. In subject 5, the endothelial image was considerably affected and was hardly distinguished by noncontact specular microscopy (Fig 2, right, second). In contrast, using contact specular microscopy, 1.5 hours after ripasudil administration CECs showed less definitive morphological change though there were some irregular cell borders. A very few dark or swollen cells were observed only sporadically and they did not show the characteristic morphology of guttae (Fig 3, right, second). These morphological changes were detected in common in all of 6 subjects. These changes resolved and CEC morphology returned to normal by 6 hours after administration in 5 of 6 subjects by specular microscopy (Figs 2 and 3, left and middle columns). In subject 5, minor guttae like dark cells was still detectable at 6 hours after administration using noncontact specular microscopy (Fig 2, right, third) but were not evident by contact specular microscopy (Fig 3, right, third). After repeated administration of ripasudil, contact specular microscopic images of CECs in all subjects showed healthy morphology prior to the final administration on day 7 (Fig 3, bottom). At the follow-up observation, no abnormal findings of CECs were detected by contact or noncontact specular microscopy in any subjects (data not shown). Serial observations of CECs by noncontact and contact specular microscopies on day 0 and day 7 revealed the morphological changes to be reversible and no morphological abnormalities were observed in the CECs.

ECD was evaluated at 6 time points and at the follow-up observation by noncontact and contact specular microscopies (Tables 1 and 2). ECD did not change throughout observation period other than 1.5 hours after ripasudil administration. At 1.5 hours, ECD was difficult to evaluate for all 6 subjects by noncontact specular microscopy and in 3 subjects by contact specular microscopy due to the blurred images with considerable morphological changes. Reliable data was obtained from subjects 1, 3 and 4 at 1.5 hours by contact specular microscopy and decreased ECD was not observed in these subjects. Moreover none of the 6 subjects showed decreased ECD at the follow-up observation.

**Table 1:** Endothelial Cell Density Examined by Noncontact Specular Microscopy Before and After the Administrations of Ripasudil in Instilled Eye. N/A = not able to be calculated or unreliable data, SD = standard deviation. Values are the number of corneal endothelial cells (/mm^2^).

Subject	Day 0			Day 7			Follow-up
	Pre^a^	1.5 hrs.	6 hrs.	0 hr^b^	1.5 hrs.	6 hrs.	
1	3252	N/A	3280	3201	N/A	3210	3175
2	2692	N/A	2583	2664	N/A	2478	2590
3	3001	N/A	2874	2974	N/A	2929	2881
4	2567	N/A	2426	2628	N/A	2613	2597
5	2384	N/A	2296	2283	N/A	2427	2614
6	2824	N/A	2702	3010	N/A	2825	2982
Mean	2786.7	-	2693.5	2793.3	-	2747.0	2806.5
SD	310.9	-	351.7	331.7	-	298.8	244.9

^a^ Before the administration of ripasudil.

^b^ Before the final administrations of ripasudil (Twelve hours after administration).

**Table 2:** Endothelial Cell Density Examined by Contact Specular Microscopy Before and After the Administrations of Ripasudil in Instilled Eye. N/A = not able to be calculated or unreliable data, SD = standard deviation. Values are the number of corneal endothelial cells (/mm^2^).

Subject	Day 0			Day 7			Follow-up
	Pre^a^	1.5 hrs.	6 hrs.	0 hr^b^	1.5 hrs.	6 hrs.	
1	3175	3135	3155	3390	3378	3378	3300
2	2646	N/A	2688	2604	N/A	2611	2545
3	3125	3115	3226	3215	3145	3165	3145
4	2283	2247	2353	2525	2506	2564	2421
5	2755	N/A	2725	2849	N/A	2933	2833
6	3058	N/A	2941	2967	N/A	2994	3096
Mean	2840.3	-	2848.0	2925.0	-	2940.8	2890.0
SD	345.3	-	326.0	337.9	-	314.7	351.5

^a^ Before the administration of ripasudil.

^b^ Before the final administrations of ripasudil (Twelve hours after administration).

**Fig 4:**
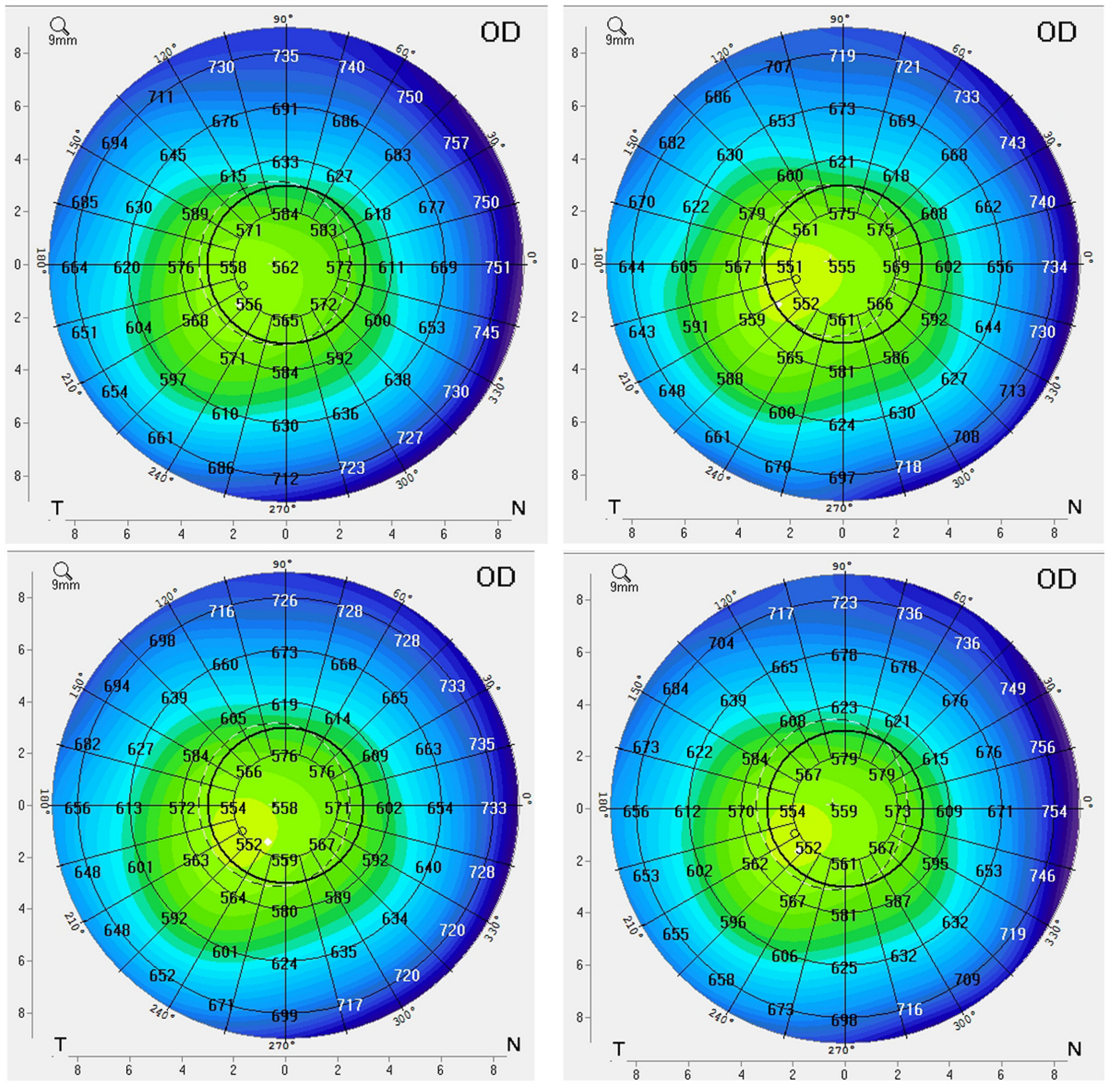
Corneal pachymetry mapping in instilled eye. Corneal pachymetry mapping of subject 4 on day 0 before administration (Top left), 1.5 hours (Top right) and 6 hours (Bottom left) after the initial administration and on day 7 prior to the final administration (Bottom right) of ripasudil in instilled eye. Throughout the observation period, there was no change of corneal thickness of the center and peripheral cornea.

**Fig 5:**
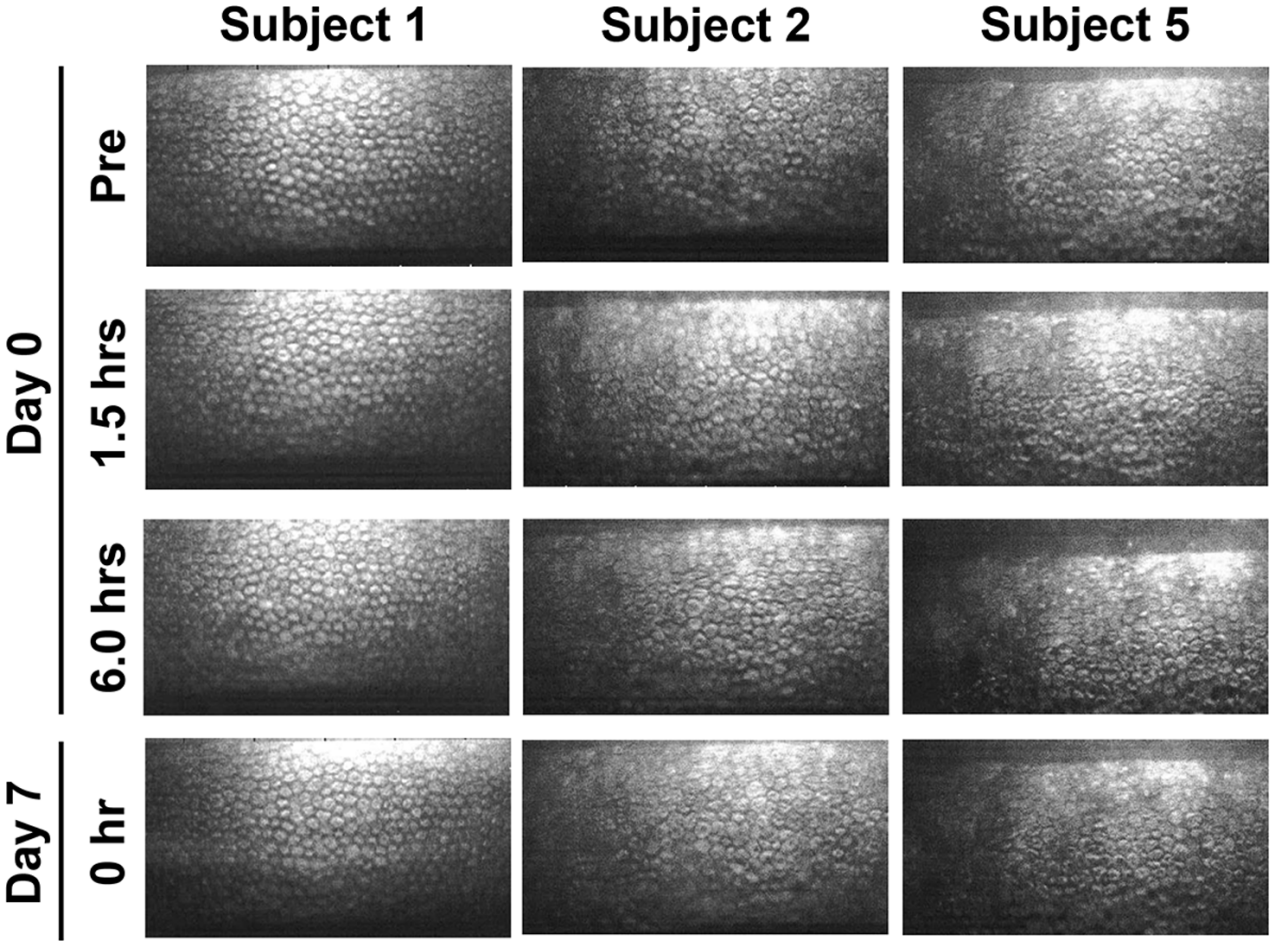
Noncontact specular microscopy images of corneal endothelium before and after ripasudil administration in non-instilled eye. In 3 of 6 typical healthy subjects, before administration (Top row), 1.5 hours after the initial administration on day 0 (Second row), 6 hours after the initial administration on day 0 (Third row), and before the final administration on day 7 (Bottom row) of ripasudil in non-instilled eye, in subject 1 (Left column), subject 2 (Middle column), and subject 5 (Right column). Throughout the observation period, there were no morphological changes of corneal endothelial cells in non-instilled eye.

**Fig 6:**
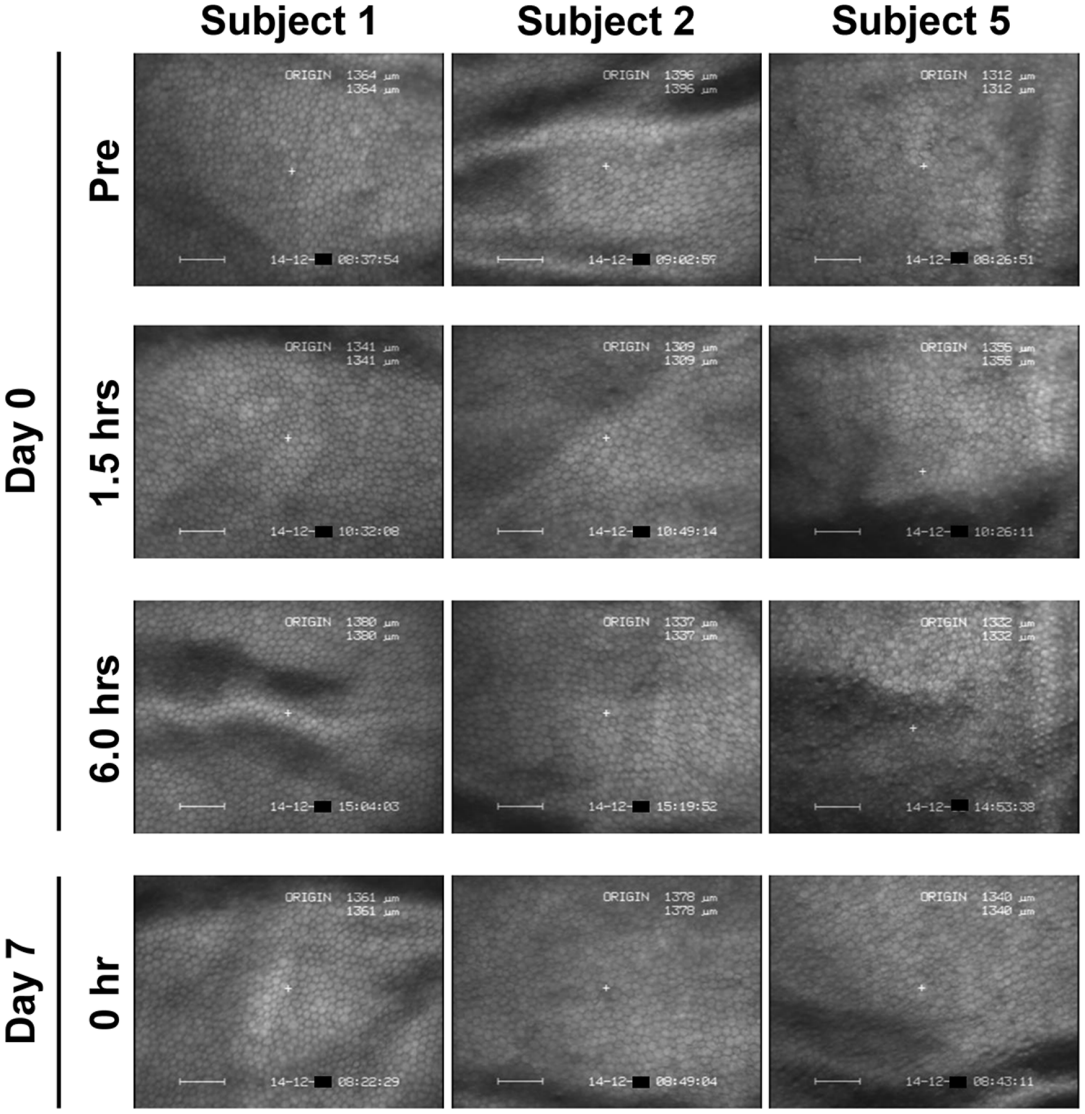
Contact specular microscopy images of corneal endothelium before and after ripasudil administration in non-instilled eye. In 3 of 6 typical healthy subjects, before administration (Top row), 1.5 hours after the initial administration on day 0 (Second row), 6 hours after the initial administration on day 0 (Third row), and before the final administration on day 7 (Bottom row) of ripasudil in non-instilled eye, in subject 1 (Left column), subject 2 (Middle column), and subject 5 (Right column). Throughout the observation period, there were no morphological changes of corneal endothelial cells in non-instilled eye.

To examine the effect of ripasudil on corneal endothelial function of healthy subjects, we evaluated CCT (Table 3) and corneal volume of the 5 mm-diameter area at the central cornea (Table 4) at 6 time points and at the follow-up observation using Pentacam Scheimpflug topography. We did not find any changes for either CCT or corneal volume for any of the 6 subjects. Corneal pachymetry mapping data obtained by Pentacam Scheimpflug topography supported the findings obtained by CCT and corneal volume (Fig 4). Best-corrected visual acuity did not change in any of the subjects eyes. In the non-instilled eye, there were no morphological changes in CECs (Figs 5 and 6), changes of ECD (Tables 5 and 6), or change in CCT (Table 7) or corneal volume (Table 8) observed in any of the subjects. The only drug related adverse events observed were mild conjunctival hyperemia, which was detected in 5 of 6 subjects (except subject 1), and resolved prior to the next administration in all cases. By slit-lamp microscopy, the cornea remained clear throughout the observation period and corneal stromal and epithelial edema was not detected in any subject. Mild guttae like endothelial reflection, corresponding to the specular microscopic image, was observed at 1.5 hours after the initial administration in 1 subject (subject 2). This finding was resolved by 6 hours.

**Table 3:** Central Corneal Thickness Before and After the Administrations of Ripasudil in Instilled Eye. SD = standard deviation. Values are the central corneal thickness (μm) examined by Pentacam Scheimpflug topography.

Subject	Day 0			Day 7			Follow-up
	Pre^a^	1.5 hrs.	6 hrs.	0 hr^b^	1.5 hrs.	6 hrs.	
1	559	550	556	558	555	555	555
2	559	575	569	564	561	561	564
3	587	575	575	568	566	575	580
4	560	554	556	558	559	554	567
5	558	553	559	554	556	555	562
6	561	559	555	561	562	561	559
Mean	564.0	561.0	561.7	560.5	559.8	560.2	564.5
SD	11.3	11.2	8.3	5.0	4.1	7.9	8.6

^a^ Before the administration of ripasudil.

^b^ Before the final administrations of ripasudil (Twelve hours after administration).

**Table 4:** Corneal Volume of 5mm Diameter Area of Central Cornea Before and After the Administrations of Ripasudil in Instilled Eye. SD = standard deviation. Values are the corneal volume of 5mm diameter area (mm^3^).

Subject	Day 0			Day 7			Follow-up
	Pre^a^	1.5 hrs.	6 hrs.	0 hr^b^	1.5 hrs.	6 hrs.	
1	12.1	11.9	12.0	12.1	12.1	12.0	12.0
2	11.9	12.2	12.1	12.1	11.9	11.9	11.9
3	12.0	12.1	12.1	12.1	11.9	12.1	12.3
4	11.9	11.7	11.8	11.8	11.8	11.7	12.0
5	11.9	11.8	12.0	11.9	11.9	11.8	12.0
6	11.9	11.9	11.8	11.9	12.0	11.9	11.9
Mean	11.95	11.93	11.97	11.98	11.93	11.90	12.02
SD	0.08	0.19	0.14	0.13	0.10	0.14	0.15

^a^ Before the administration of ripasudil.

^b^ Before the final administrations of ripasudil (Twelve hours after administration).

**Table 5:** Endothelial Cell Density Examined by Noncontact Specular Microscopy Before and After the Administrations of Ripasudil in Non-instilled Eye. SD = standard deviation. Time points indicate administrations of ripasudil in instilled eye. Values are the number of corneal endothelial cells (/mm^2^).

Subject	Day 0			Day 7			Follow-up
	Pre^a^	1.5 hrs.	6 hrs.	0 hr^b^	1.5 hrs.	6 hrs.	
1	3187	3175	3201	3300	3274	3135	3221
2	2648	2578	2517	2543	2566	2544	2626
3	3006	3074	2886	3130	3042	3036	3074
4	2459	2404	2472	2465	2437	2380	2476
5	2690	2548	2657	2507	2611	2697	2618
6	2864	2744	2873	2978	2805	2819	2955
Mean	2809.0	2753.8	2767.7	2820.5	2789.2	2768.5	2828.3
SD	263.6	308.4	274.0	361.2	317.7	288.1	296.6

^a^ Before the administrations of ripasudil in instilled eye.

^b^ Twelve hours after the administrations of ripasudil in instilled eye.

**Table 6:** Endothelial Cell Density Examined by Contact Specular Microscopy Before and After the Administrations of Ripasudil in Non-instilled Eye. SD = standard deviation. Time points indicate administrations of ripasudil in instilled eye. Values are the number of corneal endothelial cells (/mm^2^).

Subject	Day 0			Day 7			Follow-up
	Pre^a^	1.5 hrs.	6 hrs.	0 hr^b^	1.5 hrs.	6 hrs.	
1	3185	3344	3236	3247	3236	3226	3195
2	2681	2695	2710	2618	2717	2625	2558
3	3040	3289	3030	3106	3155	3125	3086
4	2381	2463	2364	2353	2488	2433	2358
5	2681	2786	2710	2865	2770	2747	2762
6	2976	2915	2778	2809	2801	2809	3067
Mean	2824.0	2915.3	2804.7	2833.0	2861.2	2827.5	2837.7
SD	295.7	344.5	299.9	323.8	282.4	300.3	333.5

^a^ Before the administrations of ripasudil in instilled eye.

^b^ Twelve hours after the administrations of ripasudil in instilled eye.

**Table 7:** Central Corneal Thickness in Non-instilled Eye. SD = standard deviation. Time points indicate administrations of ripasudil in instilled eye. Values are the central corneal thickness measured by Pentacam Scheimpflug topography (μm).

Subject	Day 0			Day 7			Follow-up
	Pre^a^	1.5 hrs.	6 hrs.	0 hr^b^	1.5 hrs.	6 hrs.	
1	552	551	549	554	557	549	551
2	556	562	563	565	562	567	555
3	565	573	575	567	571	575	572
4	559	564	557	564	559	560	562
5	551	550	553	554	551	549	553
6	552	550	554	558	551	552	556
Mean	555.8	558.3	558.5	560.3	558.5	558.7	558.2
SD	5.4	9.5	9.3	5.8	7.5	10.7	7.7

^a^ Before the administrations of ripasudil in instilled eye.

^b^ Twelve hours after the administrations of ripasudil in instilled eye.

**Table 8:** Corneal Volume of 5mm Diameter Area of Central Cornea in Non-instilled Eye. SD = standard deviation. Time points indicate administrations of ripasudil in instilled eye. Values are the corneal volume (mm^3^).

Subject	Day 0			Day 7			Follow-up
	Pre^a^	1.5 hrs.	6 hrs.	0 hr^b^	1.5 hrs.	6 hrs.	
1	11.8	11.9	11.8	11.9	12.0	11.8	11.8
2	11.8	11.9	11.9	12.0	11.9	12.0	11.7
3	12.0	12.2	12.2	12.1	12.1	12.2	12.2
4	11.9	12.0	11.8	12.0	11.8	11.9	12.0
5	11.8	11.8	11.8	11.9	11.8	11.8	11.8
6	11.6	11.6	11.7	11.8	11.6	11.6	11.7
Mean	11.82	11.90	11.87	11.95	11.87	11.88	11.87
SD	0.13	0.20	0.18	0.10	0.18	0.20	0.20

^a^ Before the administrations of ripasudil in instilled eye.

^b^ Twelve hours after the administrations of ripasudil in instilled eye.

## Discussion

This study was designed and conducted to examine the effect and safety of a selective ROCK inhibitor, ripasudil 0.4% ophthalmic solution, on CECs in healthy subjects. Six healthy subjects with healthy cornea were instilled with ripasudil 0.4% twice daily for 1 week. Considerable morphological changes in CECs were observed by noncontact specular microscopy in all subjects at 1.5 hours after ripasudil administration. Since these changes were detected only in ripasudil instilled eyes but not in non-instilled eyes, it was considered to be a phenomenon associated with ripasudil administration.

One and half hours after ripasudil administration, irregularity of cell borders with guttae like dark cells was observed in CEC images taken by noncontact specular microscope. These findings were similar to the results from previous studies in cynomolgus monkeys [18], so these corneal endothelial responses to ripasudil might be common in primates. The reason for these findings detected in the animal study was considered to be due to the reflected light of specular microscopy being scattered by CECs with morphological changes [18]. However, the actual changes in CECs were not adequately examined due to the limitation of quality of CECs observed by noncontact specular microscopy.

In this clinical study, we used specially designed, slit-scanning, wide-field contact specular microscope to analyze the effect of ripasudil on corneal endothelial morphology, and we successfully obtained clearer images than those of noncontact specular microscopy. The contact specular microscope we used in this study is a machine KSSP made by Konan Medical, Inc. This machine has a slit-scanning system and effectively reduces the light scattering by CECs. In this study, we were able to observe the morphological changes of CECs caused by ripasudil administration in detail using contact specular microscopy. We found that CECs showed indistinct cell borders with a very few dark or swollen cells but there was no findings of loss of CECs. Considerable morphological changes with multiple guttae like cells which was observed by noncontact specular microscopy was not actually detected using contact specular microscopy.

As pharmacological effects of ROCK inhibitor, cell morphological changes induced by disruption of actin microfilament bundles and impairment of focal adhesion formation were reported [4,19], and similar morphological changes of CECs might also be induced by ripasudil. Moreover, similar findings were reported from previous studies in cynomolgus monkeys by instillation of both ripasudil and Y-39983 [18], and therefore, morphological changes of CECs could be a common characteristic of ROCK inhibitors. The reason that images using contact specular microscopy were clearer than those by noncontact specular microscopy, was thought to be due to a decrease in the influence of scattering of reflected light owing to the device being in contact with the corneal surface.

Similar morphological change of CECs detected by specular microscopy was previously reported in cynomolgus monkey after the administration of H-7, serine-threonine kinase inhibitor [20]. In that paper, it was reported that CEC borders became indistinct by 1 hour after H-7 administration but cell morphology was mostly recovered by 3–5 hours and had completely recovered to normal by 24 hours without changes in ECD. H-7 is a serine-threonine kinase which decreases intraocular pressure by relaxing and expanding the trabecular meshwork and Schlemm’s canal, which is a similar mechanism as ripasudil on intraocular pressure. It is interesting that CECs of human subjects with ripasudil administration showed a similar change with that of cynomolgus monkey administered H-7. Though the mechanism of this pseudo guttae formation is yet to be elucidated, it might have occurred due to morphological changes of CECs induced by the pharmacological effect of ripasudil on actin microfilament which might lead to strongly-scattered reflected light from the specular microscopy.

In previous studies, pseudo guttae with circumscribed black zones were observed after insertion of soft contact lenses in unadapted patients and they were referred to as “endothelial bleb” [21]. The causes of endothelial blebs from wearing soft contact lenses were ascribed to several factors, including the mechanical effects of the lens, reduced corneal oxygenation, interference with carbon dioxide (CO_2_) efflux and changes in corneal pH [22]. These factors are not likely to be involved in subjects with ripasudil administration, so the mechanism of pseudo guttae formation in ripasudil instilled eyes is likely different from patients wearing soft contact lenses.

The morphological changes in CECs after ripasudil administration were confirmed to be reversible effects and CECs returned to normal prior to the next administration for all subjects. In addition, ECD, CCT, corneal volume, and best-corrected visual acuity remained stable for all subjects. We conducted post hoc Wilcoxon signed rank test for changes from before administration on Day 0 in ECD, CCT and corneal volume. No significant differences were found in the instilled eye, and significant differences were found for only 1 time point for 2 ocular examinations in the non-instilled eye; CCT before administration on Day 7 (*P* = 0.03, two-sided); and corneal volume before administration on Day 7 (*P* = 0.03, two-sided). These differences were thought to be due to random fluctuations in the non-instilled eye. Thus, no findings of cell loss in CECs might have occurred, and we assumed that the findings similar to guttae were pseudo guttae which might have occurred due to transient morphological change of CECs. Pseudo guttae formation was a transient effect of ripasudil and these changes were different from “real” guttae where accumulation of abnormal extracellular matrix is often observed in corneal endothelial diseases such as Fuchs endothelial corneal dystrophy. Additionally, 1-year clinical evaluation of ripasudil 0.4% in patients with open-angle glaucoma and ocular hypertension revealed acceptable safety profile and there were no corneal endothelial disorders reported as adverse events in the long-term study [23].

In conclusion, this study revealed that transient morphological changes identified by indistinct cell borders and appearance of pseudo guttae were observed in CECs after ripasudil administration using noncontact specular microscopy. This may induce a transient functional alteration of corneal endothelium. However, the morphological changes were mild when observed by contact specular microscopy and there were no findings of CEC loss. These morphological changes did not effect corneal thickness or ECD. The recovery of CECs was confirmed by contact specular microscopy prior to the next administration of ripasudil. These results support the safety of short-term use of ripasudil on CECs in subjects with healthy cornea. Further studies are needed in order to explore the long-term effects of ripasudil and the underlying cellular mechanism of the effect on CECs.

## Supporting Information

S1 Protocol Trial Protocol. (PDF)

S1 TREND Checklist TREND Checklist. (PDF)
