# Supplementary material for: Morphological Changes of Human Corneal Endothelial Cells after Rho-Associated Kinase Inhibitor Eye Drop (Ripasudil) Administration: A Prospective Open-Label Clinical Study
Source: PLoS One. 2015 Sep 14;10(9):e0136802. doi: 10.1371/journal.pone.0136802 (PMC4569567; doi:10.1371/journal.pone.0136802)
Supplement: S1 Protocol — (PDF) [file pone.0136802.s001.pdf]

Confidential

K-115 Ophthalmic Solution
Clinical Pharmacological Study

Effects on the cornea of healthy adults

Study Protocol

Sponsor	Kowa Company, Ltd.
Protocol No.	K-115-R-03

The information contained in this protocol is confidential and is provided to the investigator, sub-investigator, trial clinical research coordinators, other persons involved in the trial, head of the study institution, and institutional review board. It must not be disclosed to a third party without written consent of Kowa Company, Ltd.

[Contact information]
Kowa Company, Ltd.
3-4-14 Nihonbashihoncho, Chuo-ku, Tokyo 103-8433
TEL +81-3-3279-7417　FAX +81-3-3242-2270

[Out-of-hours points of contact]
Monitor supervisor: Hitoshi Kawamura (mobile phone: XXX-XXXX-XXXX)
Trial supervisor: Mitsumasa Nakamura (mobile phone: XXX-XXXX-XXXX)

Summary

Items	Contents
Study objective	The effects of K-115 ophthalmic solution (0.4%), repeatedly instilled twice daily for one week, on the cornea of healthy adults are examined after single or 1-week repeated instillation.
Methods	
Study design	Open-label study at two facilities
Usage/dosage	A single drop of K-115 ophthalmic solution (0.4%) is instilled in the right eye twice (morning and evening) daily.
Observation, Administration, and Follow-up period	Administration period: 1 week<br>(Observation period: 1 day to 4 weeks before the start of administration period; Follow-up period: 4 to 8 weeks after the end of administration period)
Number of subjects	6 subjects (the number of subjects in the administration period)

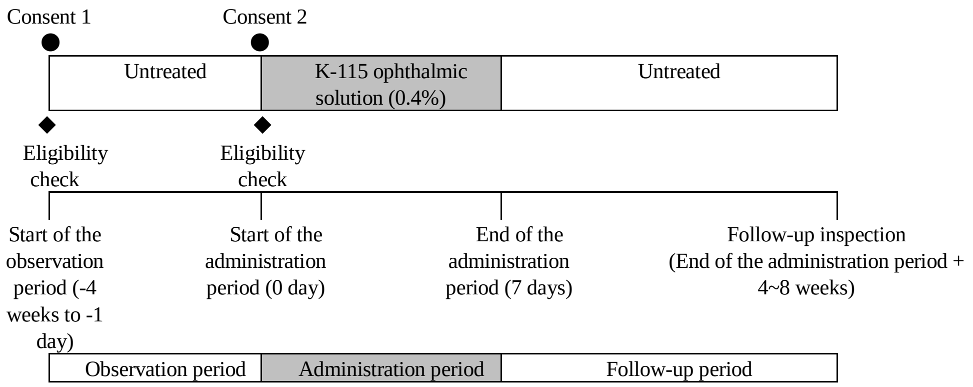

Subjects	
Inclusion criteria	The subjects included in this clinical trial met the following criteria:<br>(1) Healthy Japanese adults<br>(2) Aged 20 -65 when consent 1 was obtained

Items	Contents
Exclusion criteria	Subjects who met any one of the following criteria are excluded from this clinical trial:<br>(1) Ophthalmic (excluding mild or moderate myopia, hyperopia, astigmatism, and presbyopia) or systemic disease or abnormality in inspection at the start of the observation period<br>(2) Subjects who have undergone ophthalmological surgeries or ophthalmological laser surgeries in either eye before the beginning of the observation period<br>(3) Ophthalmic procedures in either eye before the start of the observation period;<br>However, the following ophthalmic procedures are acceptable:<br>・Chalazion puncturing, eyelash removal, and foreign matter removal from the conjunctiva<br>(4) Prohibited concomitant treatment planned between the start of the observation period and the end of the follow-up inspection<br>Prohibited concomitant treatment<br>1) All medications (including OTC drugs)<br>• With the exception of medications used for ophthalmic inspection in this clinical trial, treatments of adverse events, and treatments of accidental diseases in the observation period<br>2) Ophthalmological surgeries or ophthalmological laser surgeries<br>3) Ophthalmic procedures<br>• With the exception of chalazion puncturing, eyelash removal, and foreign matter removal from the conjunctiva as treatments for adverse events<br>4) Contact lens<br>5)Others: procedures affecting the evaluation of the investigational drug and use of medical devices

Items	Contents
	(5) < 1.0 of best corrected visual acuity in either eye in the inspection at the start of the observation period<br>(6) < 2,000 cells/mm^2 of corneal endothelial cell density in either eye by non-contact specular microscopy at the start of the observation period<br>(7) Corneal guttata in either eye by non-contact specular microscopy at the start of the observation period<br>(8) > 600-µm corneal thickness in either eye in any of two three-dimensional analyses of the anterior segment at the start of the observation period<br>(9) Subjects with a history of shock or anaphylaxis as a drug hypersensitivity reaction<br>(10) Subjects who are pregnant, breast-feeding, or expecting pregnancy<br>(11) Prescription of an investigational drug in a different clinical trial in 30 days before the start of the observation period or simultaneous participation in a different clinical trial<br>(12) Previous prescription of K-115 ophthalmic solution (excluding a placebo)<br>(13) Alcoholism or drug addiction<br>(14) Subjects judged by the investigator or sub-investigator to be inappropriate for participation in the clinical trial
Termination criteria	(1) Voluntary withdrawal<br>(2) Discontinuation for non-medical reason(s)<br>(3) Adverse events precluding the continuation of the clinical trial<br>(4) Prohibited concomitant treatments<br>Excluding treatments of adverse events and those of accidental diseases in the observation period, as determined necessary by an investigator or sub-investigator.<br>(5) Difficulties with continuing the clinical trial, as determined by an investigator or sub-investigator<br>(6) Ineligibility determined by an investigator or sub-investigator after the start of the clinical trial

Items	Contents
Safety evaluation	
Evaluation items	[Evaluation item 1]<br>Adverse events and adverse drug reactions<br>[Evaluation item 2]<br>Morphology of corneal endothelial cells<br>[Evaluation item 3]<br>Corneal endothelial cell density, corneal thickness, corneal volume, slit-lamp examination, visual acuity, and intraocular pressure
Endpoints	[Endpoint for evaluation item 1]<br>Adverse events and adverse drug reactions<br>[Endpoint for evaluation item 2]<br>Morphological abnormality of corneal endothelial cells<br>[Endpoint for evaluation item 3]<br>Endpoint A: Amounts of change at each time point after the start of the administration period<br>Endpoint B: Difference in endpoint A between the right and left eyes of the subjects [Difference between K-115 ophthalmic solution (0.4%) administration (right eye) and non-administration (left eye)]
Organization	
Sponsor	Kowa Company, Ltd.
Medical expert	Noriko Koizumi, a professor of the Department of Biomedical Engineering, Faculty of Life and Medical Sciences, Doshisha University
Trial period	From November 2014 to April 2015

Table of contents

Summary 3
Table of contents 7
1 Trial implementation system 10
1.1 Sponsor..10
1.1.1 Sponsor...10
1.1.2 Trial supervisor...10
1.1.3 Statistical analysis manager 10
1.1.4 Medical expert...10
1.2 Institutions...10
1.2.1 Investigators and institutions 10
2 Background information..11
2.1 Name of investigational drug...11
2.1.1 Development code 11
2.1.2 Generic name..11
2.1.3 Chemical name 11
3 Study objective...12
4 Study design 13
4.1 Evaluation item...13
4.1.1 Efficacy endpoint..13
4.1.2 Safety endpoint..13
4.2 Study type and design..13
4.2.1 Study type..13
4.2.2 Study design 13
4.3 Methods for randomization and double-blinding to minimize bias in clinical trials..................14
4.4 Administration method and dose of investigational drug...15
4.5 Rules for termination...16
5 Investigational product...17
5.1 Investigational product 17
5.1.1 Name of investigational drug..17
5.1.2 Dosage form/content...17
5.1.3 Stability 17
5.2 Packaging and labeling of investigational drug..17
5.2.1 Package forms 17
5.2.2 Labeling..17
5.3 Management of investigational drug..17
5.4 Randomization and blinding of investigational drug 18
6 Subject inclusion and exclusion and termination criteria...19

6.1 Inclusion criteria 19
6.2 Exclusion criteria 19
7 Treatments for subjects 21
7.1 Names of drugs, administration method/dose, administration period 21
7.2 Treatments permitted or prohibited before or during the trial 21
7.2.1 Treatments prohibited to be used concomitantly 21
7.3 Procedures to check subject’s compliance with medication and others 21
7.3.1 Confirmation of compliance 21
7.3.2 Instructions to subjects 22
7.4 Selection of subjects 23
7.5 Observation and inspection items and measuring methods and periods 23
7.5.1 Consent and subject background 25
7.5.2 Examinations and interviews 26
7.5.3 Ophthalmological examinations 27
7.5.4 Other examinations (optional) 30
7.6 Discontinuation criteria and procedures 32
7.7 Adverse events 33
7.7.1 Definition of adverse events 33
7.7.2 Investigation and recording of adverse events 33
7.7.3 Evaluation of adverse events 33
7.7.4 Measures to take in subjects after the occurrence of adverse events 35
7.7.5 Procedure for actions and reporting at the occurrence of serious adverse events 35
8 Efficacy evaluation 37
8.1 Characteristics of demographic and other standard values 37
8.1.1 Items 37
8.1.2 Calculations and statistics 37
8.2 Identification of efficacy evaluation indices 37
9 Safety evaluation 38
9.1 Identification of safety evaluation indices 38
9.2 Evaluation and analysis 38
9.2.1 Analysis of each endpoint 38
9.3 Additional analysis 39
10 Statistical analysis 40
10.1 Techniques of statistical analysis 40
10.2 Target number of subjects and its rationale 40
10.3 Level of significance and confidence coefficient 40
10.4 Termination criteria 40
10.5 Analysis sets 40

10.5.1 Efficacy analysis sets 40
10.5.2 Safety analysis set 40
10.6 Handling of the subjects and data 40
10.6.1 Subject handling 40
10.6.2 Data handling 41
10.7 Changes in the statistical analysis plan 42

1 Trial implementation system

1.1 Sponsor

1.1.1 Sponsor

Kowa Company, Ltd.

3-6-29 Nishiki, Naka-ku, Nagoya City, Aichi Prefecture 460-8625

1.1.2 Trial supervisor

Mitsumasa Nakamura

Kowa Company, Ltd.

TEL +81-3-3279-7417 FAX +81-3-3242-2270

1.1.3 Statistical analysis manager

Hideki Suganami

Kowa Company, Ltd.

TEL +81-3-3279-7388 FAX +81-3-3279-7869

1.1.4 Medical expert

Noriko Koizumi

A professor of the Department of Biomedical Engineering, Faculty of Life and Medical Sciences, Doshisha University

1.2 Institutions

1.2.1 Investigators and institutions

Dr Shigeru Kinoshita

University Hospital, Kyoto Prefectural University of Medicine

Dr Hiroshi Mikami

Heishinkai Medical Group Incorporated, OPHAC Hospital

2 Background information

2.1 Name of investigational drug

2.1.1 Development code

K-115-R

2.1.2 Generic name

Ripasudil hydrochloride hydrate

2.1.3 Chemical name

4-Fluoro-5-{[(2*S*)-2-methyl-1,4-diazepan-1-yl]sulfonyl}isoquinoline monohydrochloride dihydrate

3 Study objective

The effects of K-115 ophthalmic solution (0.4%), repeatedly instilled twice daily for one week, on the cornea of healthy adults are examined after single or 1-week repeated instillation.

4 Study design

4.1 Evaluation item

4.1.1 Efficacy endpoint

None

4.1.2 Safety endpoint

4.1.2.1 Evaluation item

- Evaluation item 1

  Adverse events and adverse drug reactions

- Evaluation item 2

  Morphology of corneal endothelial cells

- Evaluation item 3

  Corneal endothelial cell density, corneal thickness, corneal volume, slit-lamp microscopy, visual acuity, and intraocular pressure

4.1.2.2 Endpoints

- Endpoint for evaluation item 1

  Adverse events and adverse drug reactions

- Endpoint for evaluation item 2

  Morphological abnormality of corneal endothelial cells

- Endpoints of evaluation item 3

  Endpoint A: Amounts of change of evaluation item 3 at each time point relative to those before the start of the administration period

  Endpoint B: Difference in endpoint A between the right and left eyes of the subjects [Difference between K-115 ophthalmic solution (0.4%) administration (right eye) and non-administration (left eye)]

4.2 Study type and design

4.2.1 Study type

Clinical pharmacological study

4.2.2 Study design

This open-label clinical trial is implemented at two facilities.

The design of this trial is summarized in Figure 4-1. The observation period is defined as a period from the start of observation to the start of administration. The administration period is defined as a period of repeated administration twice daily for a week, following the single instillation of K-115 ophthalmic solution (0.4%). The follow-up period is defined as a period until the conditions of corneal endothelial cells in the absence of treatment are confirmed by inspection after 6-hour instillation at the

end of the administration period.

In Heishinkai Medical Group Incorporated OPHAC Hospital, the inspection at the start of the observation period is conducted after consent (consent 1) is obtained. At the start of the observation period (from -4 weeks to -1 day), subject eligibility is checked based on inclusion and exclusion criteria. Subsequently, consent (consent 2) is obtained from eligible subjects at University Hospital, Kyoto Prefectural University of Medicine, and eligibility results are checked at OPHAC Hospital. The eligible subjects were examined in the morning before instillation as inspection before the start of the administration period, followed by single instillation of K-115 ophthalmic solution (0.4%) in the right eye, and were inspected at 1.5 and 6 hours after instillation in the morning. Subsequently, a K-115 ophthalmic solution (0.4%) was repeatedly instilled in the right eye twice daily (in the morning and evening) for 1 week ,followed by inspection at the end of the administration period (before instillation and at 1.5 and 6 hours after instillation). Follow-up inspection is conducted 4-8 weeks after the last day of investigational drug administration. In both institutions, treatments, additional inspections, and follow-up inspections can be conducted for subjects who developed adverse events.

The target number of subjects is 6 (the number of subjects to be introduced at the administration period).

[Rationale]

Considering that the subjects are healthy adults, the subjects were screened at OPHAC Hospital. The administration and later periods were implemented at University Hospital, Kyoto Prefectural University of Medicine which is equipped with contact specular microscopes, which allow the examination of the detailed conditions of the corneal endothelium. To ensure the safety of the subjects at both institutions, treatments, additional inspections, and follow-up inspection could be conducted for subjects with adverse events.

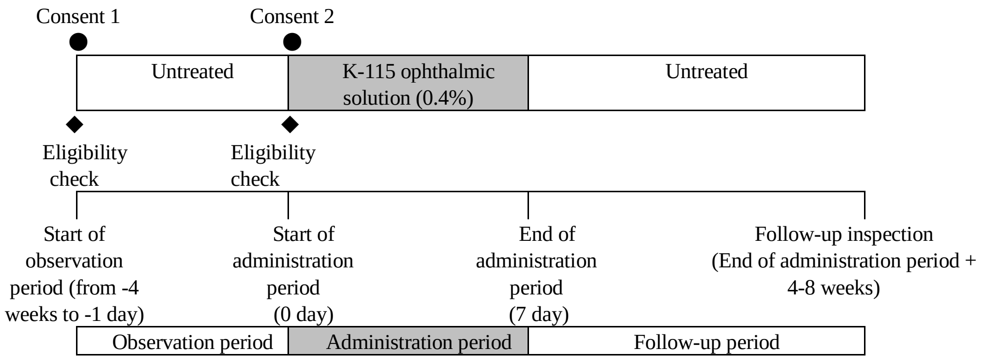

Figure 4-1. Outline of the study design

4.3 Methods for randomization and double-blinding to minimize bias in clinical trials

This clinical trial was neither randomized nor blinded.

4.4 Administration method and dose of investigational drug

A single drop of K-115 ophthalmic solution (0.4%) was instilled twice daily in the right eye (in the morning and evening).

4.5 Rules for termination

No termination criteria related to evaluations such as interim analysis are set in this study.

5 Investigational product

5.1 Investigational product

5.1.1 Name of investigational drug

K-115 ophthalmic solution 0.4%

5.1.2 Dosage form/content

The K-115 ophthalmic solution is a colorless or pale yellow transparent liquid.

Its concentration in this clinical trial is 0.4%. The dosage form and contents of the investigational drug are shown in Table 5-1.

Table 5-1. Dosage form and contents of the investigational drug

Investigational drug	Dosage form and contents
K-115 ophthalmic solution (0.4%)	An ophthalmic solution containing 4.0 mg of free K-115 in 1 mL

A benzalkonium chloride solution is contained as a preservative.

5.1.3 Stability

K-115 ophthalmic solution (0.4%) was stable in long-term (25°C/40% RH) (24 months) and accelerated (<40°C/25% RH) (6 months) tests.

Thus, the storage conditions and shelf life of K-115 ophthalmic solution (0.4%) are determined to be "at room temperature for 36 months (24 months ensured by long-term data according to ICHQ1E + 12 months)."

5.2 Packaging and labeling of investigational drug

5.2.1 Package forms

K-115 ophthalmic solution (0.4%) is packaged into two 5-mL polypropylene eye dropper bottles for a single dosage.

5.2.2 Labeling

The labels on the eye drop containers and boxes show the following items:

Eye drop container: Objective (clinical trial), investigational drug name, development code, lot number, storage method, and sponsor's name and address

Box: Objective (clinical trial), investigational drug name, protocol number, development code, lot number, storage method, and sponsor's name and address

Expiration date is described in separately created investigational drug management procedures.

5.3 Management of investigational drug

The sponsor specifies the details of the investigational drug management procedures in the separately created investigational drug management protocol to be delivered to the implementing

medical institutions.

After confirming the completion of preparations at the implementing medical institutions and the contract execution of the clinical trial, the sponsor delivers the investigational drug to the implementing medical institutions.

The investigational drug manager manages the investigational drug according to the investigational drug management procedures and the protocols of the implementing medical institutions.

After the completion of the clinical trial, the investigational drug manager submits remaining investigational drug and a copy of investigational drug management table to the sponsor. At that time, the investigational drug manager should protect personal information.

5.4 Randomization and blinding of investigational drug

This clinical trial was neither randomized nor blinded.

6 Subject inclusion and exclusion and termination criteria

Subjects from whom consent was obtained at OPHAC Hospital and University Hospital, Kyoto Prefectural University of Medicine and who met all the following inclusion criteria and did not meet any of the following exclusion criteria were included in this clinical trial.

6.1 Inclusion criteria

Subjects who met all the following criteria were included in this clinical trial:

(1) Healthy Japanese adults

(2) Aged 20-65 when consent 1 was obtained.

[Rationale]

(1) To obtain unbiased subject backgrounds, factors that affect the cornea, besides the investigational drug, were excluded.

(2) Subjects aged 20 years or older were included because written consent is legally effective above this age, and those aged less than 65 were included to exclude the elderly because the number of normal corneal endothelial cells decreases with age [6)].

6.2 Exclusion criteria

Subjects who met any one of the following criteria are excluded from this clinical trial:

(1) Ophthalmic (excluding mild or moderate myopia, hyperopia, astigmatism, and presbyopia) or systemic disease or abnormality in inspection at the start of the observation period

(2) Subjects who have undergone ophthalmological surgery or ophthalmological laser surgery in either eye before the beginning of the observation period

(3) Ophthalmic procedures in either eye before the start of the observation period;
However, the following ophthalmic procedures are acceptable:
・Chalazion puncturing, eyelash removal, and foreign matter removal from the conjunctiva

(4) Prohibited concomitant treatment (See 7.2.1 Prohibited concomitant treatments) planned between the start of the observation period and the end of the follow-up inspection

(5) <1.0 of best corrected visual acuity in either eye in the inspection at the start of the observation period

(6) < 2,000 cells/mm^2 of corneal endothelial cell density in either eye by non-contact specular microscopy at the start of the observation period

(7) Corneal guttata in either eye by non-contact specular microscopy at the start of the observation period

(8) >600-μm corneal thickness in either eye in any one of two three-dimensional analyses of the anterior segment at the start of the observation period

(9) Subjects with a history of shock or anaphylaxis as a drug hypersensitivity reaction

(10) Subjects who are pregnant, breast-feeding, or expecting pregnancy

(11) Prescription of the investigational drug in a different clinical trial within 30 days before the start of the observation period or simultaneous participation in a different clinical trial

(12) Subjects previously administered K-115 ophthalmic solution (excluding a placebo)

(13) Alcoholism or drug addiction

(14) Subjects judged by the investigator or sub-investigator to be inappropriate for participation in the clinical trial

[Rationale]

(1)(5)(9) Ensuring the safety of the subjects.

(2)-(4) Excluding effects on the evaluation of investigational drug effects on the cornea.

(6)-(8) Excluding subjects with corneal abnormalities because of effects on the evaluation of investigational drug effects on the cornea.

(10) Ensuring the safety of the subjects and excluding the effects of the investigational drug on the fetus because of its possible migration into the breast milk.

(11) Considering the ethical issues of the subjects and ensuring the safety of the subjects.

(12) Excluding potential biases in selecting subjects from those with results obtained by administering K-115 ophthalmic solution.

(13) Excluding difficulties in managing the subjects.

(14) Allowing the investigator or sub-investigator to make judgments in consideration of other general factors.

7 Treatments for subjects

7.1 Names of drugs, administration method/dose, administration period

Investigational product: K-115 ophthalmic solution

Administration method/dose: A single drop is instilled into the right eye twice daily (in the morning and evening).

Scheduled times of instillation: 9:30, 21:30

The investigational drug is instilled at 21:30 (±30 minutes) on the night before visit at the end of the administration period. On the day of visit, the drug is instilled after the end of inspection before instillation in the morning and at 1.5 hours after the instillation.

Administration period: 1 week (in the mornings at the start and end of the administration period)

7.2 Treatments permitted or prohibited before or during the trial

7.2.1 Treatments prohibited to be used concomitantly

The following treatments are prohibited between the start of the administration period and the end of the follow-up period. The clinical trial is discontinued when a prohibited concomitant treatment is initiated between the start of the administration period and the end of the follow-up period.

(1) All medications (including OTC drugs)

- With the exception of medications used for ophthalmic inspection in this clinical trial, treatments of adverse events, and treatments of accidental diseases in the observation period.

(2) Ophthalmological surgeries or ophthalmological laser surgeries

(3) Ophthalmic procedures

- With the exception of chalazion puncturing, eyelash removal, and foreign matter removal from the conjunctiva as treatments for adverse events

(4) Contact lens

(5) Others: procedures affecting the evaluation of the investigational drug and use of medical devices

[Rationale]

Excluding effects on the evaluation of investigational drug effects on the cornea.

7.3 Procedures to check subject’s compliance with medication and others

7.3.1 Confirmation of compliance

The subjects shall record the daily instillation of the investigational drug during the administration period and the instillation time on the night before visit at the end of the administration period (whenever possible if discontinued).

The investigator, sub-investigator, or clinical research coordinators check the instillation status by referring subjects’ diary and recorded it according to "7.5.2.1 Investigational drug instillation status."

7.3.2 Instructions to subjects

The investigator, sub-investigator, or clinical research coordinators gave the following instructions to the subjects:

(1) During the administration period, administer the investigational drug as instructed. Between the start of the observation period and the follow-up inspection, discuss and report in advance the use of drugs other than the investigational drug and the implementation of treatment.

(2) After instillation of the investigational drug, close the eyelids slowly and compress the tear sac. Wipe off the drug spilled around the eye while carefully preventing contact with the eyeball.

(3) Record the daily instillation of the investigational drug during the administration period and instillation time on the night before visit at the end of the administration period (whenever possible if discontinued) and bring the diary at visit.

(4) Do not instill the investigational drug in the morning on the day of visit at the end of the administration period, but instill it at the implementing medical institutions during the visit.

(5) If instillation of the investigational drug is forgotten, instill one dose as noticed. At that time, if the next instillation should be conducted in a short time, do not instill the missed dose, but instill the next dose as planned (do not instill two doses, including the missed dose, at the same time).

(6) On the day of visit at the end of the administration period or at the discontinuation visit and bring the investigational drug prescribed at the time of previous visit.

(7) Abstain from drinking and eating too much and excessive exercise, although permitted to go out during the waiting time of inspection on the day of visit. If going out, return at a specified time, and take a rest before the inspection.

(8) Between the start of the administration period and the follow-up inspection, abstain from smoking on the night before visit and until the end of all inspections on the day of visit.

(9) If an ophthalmic solution or ointment is applied at the same instillation time as the investigational drug to treat adverse events between the start of the observation period and the end of the follow-up period, instill the investigational drug first and, at least five minutes later, administer the concomitant drug.

(10) Report any symptom if noticed between the start of the observation period and the follow-up inspection.

(11) Report any visit to a different medical institution between the start of the observation period and the follow-up inspection. Report any emergency visit to a different medical institution after the visit. In either case, inform physicians about the participation in the clinical trial.

(12) Do not wear contact lenses between the start of the observation period and the follow-up inspection

(13) Make a hospital visit at specified time between the start of the observation period and the follow-up inspection. If you cannot visit out of necessity, report it in advance.

(14) Subjects capable of pregnancy shall take contraceptive measures after deciding to participate in the clinical trial.

(15) Do not participate in any other clinical trial between the start of the observation period and the follow-up inspection.

(16) Follow the instructions of the investigator, sub-investigator, or clinical research coordinators between the start of the observation period and the follow-up inspection.

(17) Do not discuss the study information obtained while participating in this clinical trial, to third parties.

7.4 Selection of subjects

After obtaining consent 1, the investigator or sub-investigator of OPHAC Hospital shall give identification codes to the subjects in order of consent obtainment. If consent is obtained simultaneously from multiple subjects, identification codes are given in ascending order of age based on their dates of birth.

The investigator or sub-investigator checks subject backgrounds, diagnoses, diagnostic interviews, and inclusion/exclusion criteria and ophthalmologic and other inspection as needed at the start of the observation period to confirm eligibility. The copies of the records (medical charts) of the eligible subjects are sent to University Hospital, Kyoto Prefectural University of Medicine.

The investigator or sub-investigator of University Hospital, Kyoto Prefectural University of Medicine obtains consent 2 from the eligible subjects at the start of the administration period, checks their eligibility based on the records (medical charts) at OPHAC Hospital, and starts the administration period by prescribing K-115 ophthalmic solution (0.4%).

7.5 Observation and inspection items and measuring methods and periods

The investigator, sub-investigator, or clinical research coordinators conducts medical interviews and inspection (Table 7-1). As a rule, the inspection methods (inspection equipment and conditions) at the specified hospital visit (at the start and end/discontinuation of the administration period and at follow-up inspection) in the administration period and later shall not be changed throughout the clinical trial.

Additional inspection shall be conducted irrespective of the specified implementation period, if it is considered to be necessary based on medical examination, interview, and inspection results, in order to ensure the safety of the subjects.

The subjects shall make four hospital visits: at the start of the observation and administration periods, at the end/discontinuation of the administration period, and at follow-up inspection.

If the date of hospital visit specified by the protocol differs from the actual date of visit, adjust the date of visit within the acceptable range in Table 7-2.

Table 7-1 Observation and inspection schedule

	Start of the observation period	Start of the administration period	End of the administration period/discontinuation	Follow-up inspection
Obtaining consent	○※1	○※1		
Survey of subject background	○	○		
**Medical examination and interview**				
State of instillation of the investigational drug			○	
Concomitant drug	←——	————	————	——→
Subjective symptoms (ophthalmic/systemic)	←——	————	————	——→
Objective symptoms (ophthalmic/systemic)	←——	————	————	——→
Adverse events		←——	————	——→
**Ophthalmic inspection**				
Morphological observation of corneal endothelial cells	○	③	③※2	○
Measurement of corneal endothelial cell density	○	③	③※2	○
Examination of three-dimensional analyser for the anterior segment	②	③	③※2	○
Slit-lamp microscopy	○	③	③※2	○
Tonometry	○	○※3	○※3	
Vision test	○	○	○	
**Others**				
Measurement of blood pressure and pulse rate	△			
Laboratory test	△			
Pregnancy test (if applicable)	△			

※1: Before the start of the observation and administration periods
※2: Measured once on discontinuation
※3: Measured before instillation in the morning
② : Measured twice
③ : Measured three times (once each before instillation in the morning and at 1.5 and 6 hours after instillation)
△ : No inspection is needed if eligibility can be confirmed from other data.
Inspection results are not collected in case report forms.

Table 7-2. Acceptable ranges of visit date

Evaluation periods	Acceptable ranges
Start of the observation period	<4 weeks of the observation period
Start of the administration period	0 days

End of the administration period	7 ± 1 days after the start of the administration period (Day 0)
Follow-up inspection	28~56 days after the final administration of the investigational drug

7.5.1 Consent and subject background

7.5.1.1 Obtaining consent

After execution of the study contract between the institution and sponsor, the investigator, sub-investigator, or clinical research coordinators delivers the consent form and accompanying explanatory document to subjects who have expressed their wish to participate of their own free will, sufficiently explains the contents of this study, and obtains written consent from the subject in person to participate in the trial. The trial clinical research coordinators can provide supplementary explanation while obtaining consent. In this clinical trial, the inspection at the start of the observation period is conducted at OPHAC Hospital, and the inspection at the start and end/discontinuation of the administration period and the follow-up inspection are conducted at University Hospital, Kyoto Prefectural University of Medicine. Consent is obtained at each implementing institution.

7.5.1.2 Review of the subjects' background

The investigator or sub-investigator obtains written consent, and examines and records subject background (Table 7-3).

[Time of implementation]

At the start of the observation and administration periods

Table 7-3. Survey items of subject backgrounds

Background information	Surveys and records
Date of birth	Date of birth at the start of the observation period are surveyed.
Sex	Investigation of the subject's sex
State of consultation with other departments/hospitals *[1]	The presence or absence of periodic medical examination and continued medication are surveyed.
Disease history *[1]	All diseases and symptoms that affect the subjects at the start of or during the observation period are surveyed.<br>If any, disease names and symptoms, the left or right eye for eye diseases, and outcomes at the start of the administration period are recorded.
History of surgery or laser surgery of the eye	History of the eye surgery described in the left, conducted by the start of the observation period, is surveyed. If any, it is recorded.
History of treatment using ophthalmic procedures	History of treatment using ophthalmic procedures, conducted by the start of the observation period, is surveyed. If any, treatment, left or right eye, and date of the latest treatment after the start of the observation period are recorded.

*[1] Excluding myopia, hyperopia, astigmatism, and presbyopia

7.5.2 Examinations and interviews

7.5.2.1 State of instillation of the investigational drug

At the start of the administration period, the investigator, sub-investigator, or clinical research coordinators records the time of instillation in the morning. At the end of administration period, the investigator, sub-investigator, or clinical research coordinators confirm that no drug was instilled in the morning on the day of visit. The investigational drug is instilled between the predose inspection and the 1.5 hours postdose inspection, and the investigator, sub-investigator, or clinical research coordinators record the instillation time. The number of instillations of investigational drug in the administration period and the time of instillation on the night before visit at the end of the administration period are checked on the subjects' diary. If instillation is discontinued before the end of the administration period, the number of instillations by the day before visit and instillation time if any on the night before the visit are recorded. The diary records the presence or absence of instillation in the right eye between the evening on the day of the initial visit at the start of the administration period and on the night before the final visit, and instillation time (if any on discontinuation) on the night before visit at the end of the administration period.

[Time of implementation]

At the start and end/discontinuation of the administration period

7.5.2.2 Concomitant drugs

The investigator, sub-investigator, or clinical research coordinators records the presence or absence of concomitant drugs, including those prescribed at other departments and hospitals, drug names, routes of administration, dosages, and administration periods. Concomitant drugs used between the start of the observation period and the follow-up inspection and those used to treat adverse events, excluding ophthalmic solutions (e.g., anesthetics, corneal protectants, and mydriatics) used for ophthalmological inspection in the clinical trial, were recorded.

[Times of implementation]

All days of visit

7.5.2.3 Symptoms (ocular/whole body) and signs (ocular/whole body)

The investigator or sub-investigator conducts medical interviews regarding ophthalmic and systemic subjective symptoms, examines ophthalmic and systemic objective symptoms, and records matters to be noted.

[Times of implementation]

All days of visit

7.5.3 Ophthalmological examinations

7.5.3.1 Morphological observation of corneal endothelial cells and measurement of corneal endothelial cell density

Corneal endothelial cells in the central cornea of both eyes are photographed using contact and non-contact specular microscopes, and the time of photographing and the morphological findings of the corneal endothelial cells are recorded. Also, corneal endothelial cells are photographed and corneal endothelial cell densities are determined. The image and output data are submitted to the sponsor on electronic media. The electronic data are stored separately from the case report forms.

At the start of the observation period, only a non-contact specular microscopy is used. To examine the detailed conditions of the corneal endothelium, a contact specular microscopy for research is used. Before microscopy, the subjects are topically anesthetized.

[Time of implementation]

At the start of the observation period:

No specific time regimen is required

At the start and end of the administration period:

Before instillation of the investigational drug in the morning

1.5 hours after instillation of the investigational drug

6 hours after instillation of the investigational drug

At discontinuation and follow-up inspection:

No specific time regimen is required

[Tolerated ranges]

At the start and end of the administration period:

1.5 hours and ±30 minutes after instillation of the investigational drug

6 hours and ±30 minutes after instillation of the investigational drug

7.5.3.2 Examination of three-dimensional analyser for the anterior segment

Corneal thickness and corneal volume, etc are measured for both eyes using a three-dimensional analyser for the anterior segment, and the measurement time and the corneal thickness of the center of the pupils are recorded. The image and output data are submitted to the sponsor on electronic media. The electronic data are stored separately from the case report forms.

At the start of the observation period, the measurement was conducted twice on the same day to adjust for intrasubject variation.

[Time of implementation]

At the start of the observation period:

No specific time regimen is required

At the start and end of the administration period:

Before instillation of the investigational drug in the morning

1.5 hours after instillation of the investigational drug

6 hours after instillation of the investigational drug

At discontinuation and follow-up inspection:

No specific time regimen is required

[Tolerated ranges]

At the start and end of the administration period:

1.5 hours and ±30 minutes after instillation of the investigational drug

6 hours and ±30 minutes after instillation of the investigational drug

7.5.3.3 Slit-lamp microscopy

The cornea, conjunctiva, anterior chamber, lens, and iris are examined in both eyes using a slit-lamp microscope, and the findings and their severity are recorded. Fluorescein staining is performed for corneal examination. The severity of findings is evaluated according to the criteria shown in Tables 7-4 and 7-5.

[Time of implementation]

At the start of the observation period:

No specific time regimen is required

At the start and end of the administration period:

Before instillation of the investigational drug in the morning

1.5 hours after instillation of the investigational drug

6 hours after instillation of the investigational drug

At discontinuation and follow-up inspection:

No specific time regimen is required

[Tolerated ranges]

At the start and end of the administration period:

1.5 hours and ±30 minutes after instillation of the investigational drug

6 hours and ±30 minutes after instillation of the investigational drug

Table 7-4 Evaluation criteria for slit-lamp microscopy findings (other than conjunctival hyperemia)

Severity	Contents
−	No finding, or very slight findings, which are physiologic changes
+	Mild findings
2+	Moderate findings (affecting activities of daily living)
3+	Severe findings (making activities of daily living difficult)

Table 7-5 Evaluation criteria for conjunctival hyperemia (sites: palpebral conjunctiva, bulbar conjunctiva)

Severity	Contents
−	No finding, or very slight findings, which are physiologic changes
+	Dilation of several blood vessels
2+	Dilation of many blood vessels or vasodilation in the limbal region
3+	Vasodilation over the entire conjunctiva

7.5.3.4 Tonometry

Intraocular pressure is measured for both eyes using a non-contact tonometer. The measurement is conducted several times for each eye, and mean intraocular pressure and measurement time are recorded.

[Time of implementation]

At the start of the observation period, at discontinuation:

No specific time regimen is required

At the start and end of the administration period:

Before instillation of the investigational drug in the morning

7.5.3.5 Visual acuity test

The best corrected visual acuity is measured in both eyes, and the results are recorded.

[Time of implementation]

At the start of the observation period, at the start and end of the administration period, at discontinuation:

No specific time regimen is required

7.5.4 Other examinations (optional)

The investigator or sub-investigator conducts the following inspection when he/she considered it necessary to confirm the exclusion criteria (1) and (10), although it does not need to be implemented when the eligibility can be determined based on the previous inspection results, medical certificates, and interview results. Data used as the grounds for subject eligibility are kept in medical records, and no data are collected from case report forms.

7.5.4.1 Measurement of blood pressure/heart rate

The subjects are allowed to rest for five minutes or longer, in order to measure blood pressure (systolic and diastolic blood pressure) and pulse rates in a sitting position.

[Time of implementation]

At the start of the observation period

7.5.4.2 Laboratory tests

To test the items in Table 7-6, about 8 mL of blood and about 10 mL of urine are collected.

[Time of implementation]

At the start of the observation period

Table 7-6 Items and contents of laboratory tests

Item	Contents
Hematology	WBC, RBC, Hb, Hct, Plt
Blood chemistry	Urea nitrogen, uric acid, creatinine, AST, ALT, γ-GTP, LDH, ALP, Na, K, Cl
Urinalysis	Urobilinogen qualitative, occult blood, glycosuria qualitative, proteinuria qualitative

7.5.4.3 Pregnancy test

Pregnancy testing is performed in the subjects who may become pregnant, and the results are recorded. About 10 mL/time of blood is sampled. Urine samples for laboratory tests can also be used for clinical inspection if it is conducted at the start of the observation period.

[Time of implementation]

At the beginning of the observation period

7.6 Discontinuation criteria and procedures

The trial is terminated in the subjects who meet the following criteria.

(1) When the subject drops out of his/her own free will

(2) When the subject has become unable to continue the trial for non-medical reasons

(3) When the continuation of the trial is difficult due to the occurrence of adverse events

(4) When a treatment the concomitant use of which is prohibited has been performed

- Treatments of adverse events and those of accidental diseases in the observation period are allowed, as needed by the investigator or sub-investigator.

(5) When the continuation of the trial has been judged to be difficult by the investigator or sub-investigator

(6) When the patient has been judged after the beginning of this study to be inappropriate as a subject by the investigator or sub-investigator

［Procedures］

As a rule, when the clinical trial is discontinued after the administration of the investigational drug, all possible evaluations and inspections are conducted to ensure the safety of the subjects. For the observation and inspection items at this time, see “7.5 Observation and inspection items and measuring methods and periods.”

When the clinical trial is discontinued for safety reasons, such as adverse events, the investigator or sub-investigator shall take appropriate measures and conducts a follow-up survey until symptoms (laboratory values) recovered to the levels before the start of the clinical trial or neither treatment nor follow-up is needed.

In addition, the presence or absence of discontinuation, date of deciding discontinuation, date of the final administration of the investigational drug, and follow-up results are recorded in the case report form.

If the subject does not make a hospital visit or does not complete an evaluation/inspection or a follow-up survey for some reasons, the subject’s conditions are checked, and the date of inspection, procedures, the reasons for the discontinuation, and the courses after the final visit (observation) are recorded.

7.7 Adverse events

7.7.1 Definition of adverse events

All medical events undesirable to the subjects such as new symptoms/signs that have appeared after the administration of the investigational drug while no symptom/sign was observed before the administration, as well as exacerbation of symptoms/signs observed from before the administration of the investigational drug are regarded as adverse events. The judgments about adverse events are unrelated to their causal relationship with the investigational drug.

7.7.2 Investigation and recording of adverse events

The investigator or sub-investigator makes judgments about adverse events on the basis of the results of evaluation of appreciable findings, contents and severity of abnormalities, or abnormal changes noted by interviews, examinations, ophthalmological examinations, blood pressure/heart rate measurements, and clinical laboratory tests.

Concerning the symptoms/signs regarded as adverse events, the investigator or sub-investigator records the contents and sites, date of appearance, date of outcome or its observation, pattern of occurrence (conjunctival hyperemia alone), evaluations of adverse events (severity, seriousness, relationship with the investigational drug), state of administration of the investigational drug (continued, suspended, terminated, completed), treatments required, outcome, and reasons for excluding the relationship with the investigational drug.

7.7.3 Evaluation of adverse events

7.7.3.1 Evaluation of severity

The severity of adverse events is evaluated according to the criteria shown in Table 7-7. The severity of adverse events concerning the blood pressure/heart rate, hematology, blood chemistry, and urinalysis is not evaluated.

Table 7-7 Criteria for the evaluation of severity of adverse events

Severity	Contents
1：Mild	No effect on activities of daily living
2：Moderate	Interfering with activities of daily living
3：Severe	Making activities of daily living impossible

7.7.3.2 Evaluation of seriousness

The seriousness of adverse events is evaluated according to the criteria shown in Table 7-8.

Table 7-8 Criteria for the evaluation of seriousness

Seriousness	Contents
1 : Serious	The following among all undesirable medical events that have occurred after the administration of the investigational drug<br>a. Those that lead to death

	b. Those that threaten life<br>c. Those that require hospitalization or prolongation of hospitalization for the treatment<br>d. Those that cause lasting or marked disability or functional impairment<br>e. Those that cause congenital abnormalities<br>f. Serious based on the above criteria a-e.
2 : Not serious	Other than those above

7.7.3.3 Evaluation of the relationship with the investigational drug

The relationships of adverse events with the investigational drug are classified according to the criteria shown in Table 7-9. Of the adverse events, those the causal relationships of which with the investigational drug cannot be excluded ("3. possibly related", "4. probably related", and "5. related") are regarded as adverse drug reactions.

Table 7-9. Evaluation criteria of relationship with the investigational drug

Relationship	Contents
1 : Unrelated	There are rationale reasons other than the investigational drug, such as the effects of concomitant drugs and the conditions of the subjects.
2 : Unlikely to be related	Considering the properties of the investigational drug and adverse events, the relationship with the investigational drug can be denied for the following reasons:<br>・No relationship is noted between the administration period of the investigational drug and the time of adverse event development, although no causes, other than the investigational drug, are specified.<br>・The administration of the investigational drug is continued and adverse events are reduced without treatment. Or adverse events are not reduced by discontinuing administration, or no symptoms recur after readministration.
3 : Possibly related	・When a relationship is noted between the administration period of the investigational drug and the time of adverse event development, a relationship with the investigational drug cannot be denied for the following reason:<br>The effects of the investigational drug cannot be denied, although causes other than the investigational drug, such as the effects of concomitant drugs and the conditions of the subjects, are possible.
4 : Probably related	When a relationship is noted between the administration period of the investigational drug and the time of adverse event development, a relationship with the investigational drug is suspected for the following reasons:<br>・Adverse events reportedly result from the pharmacological effects of investigational or analogous drugs in the clinical trial although causes other than the investigational drug are possible.<br>・Adverse events are alleviated by discontinuing administration.

5：Related	When a relationship is noted between the administration period of the investigational drug and the time of adverse event development, a relationship with the investigational drug can be rationally explained by the following reasons:<br>・No causes of adverse events, other than the investigational drug, are possible. In addition, adverse events reportedly result from the pharmacological effects of investigational or analogous drugs in the clinical trial. Thus, the investigational drug is identified as a cause.<br>・Symptoms recur after readministration.

7.7.3.4 Evaluation of the outcome

The outcomes of adverse events are classified according to the criteria shown in Table 7-10.

Table 7-10 Evaluation criteria for the outcome

Outcome	Contents
1：Resolution	The adverse event is judged to have been resolved
2：Alleviation	The adverse event is judged to show a tendency to resolution
3：Unresolved	The adverse event is judged not to show a tendency to resolution
4：Resolution with sequelae	Functional impairment interfering with activities of daily living is observed despite resolution of the adverse event
5：Death	A relationship of the adverse event with death is present or cannot be excluded
6：Unclear	The outcome cannot be checked for reasons such as the absence of the subject's visit

7.7.4 Measures to take in subjects after the occurrence of adverse events

When adverse events have occurred, the investigator, sub-investigator, or clinical research coordinators performs treatment and follow-up until the adverse event is resolved or treatment or follow-up is judged to be unnecessary for medically valid reasons. Also, if the investigator or sub-investigator judges it to be necessary, additional examinations, follow-up examinations, and appropriate treatments are performed after explanation to the subject to ensure the subject's safety.

If adverse events have occurred, instillation of the investigational drug is terminated, when necessary. After termination of instillation of the investigational drug (decision to terminate the trial) by the investigator or sub-investigator, instillation is not resumed even when the symptom has been alleviated.

If the observed adverse event is serious, "7.7.5 Procedures for actions and reporting at the occurrence of serious adverse events" is followed.

7.7.5 Procedure for actions and reporting at the occurrence of serious adverse events

If a serious adverse event has occurred, the investigator, sub-investigator, or clinical research coordinators promptly performs appropriate treatments to ensure the subject's safety and makes a report by the following procedure regardless of its causal relationship with the investigational drug.

After the investigator recognizes adverse event development, he/she contacts the sponsor via telephone or FAX within 24 hours, and submits a report on the serious adverse events within five days. In addition, the investigator reports the serious adverse events to the director of the implementing medical institution according to the standard operating procedures of the institution.

The investigator makes an additional report on the serious adverse events if requested by the director of the implementing medical institution, the institutional review board, or the sponsor.

[Contact information]
Kowa Company, Ltd.
3-4-14 Nihonbashihoncho, Chuo-ku, Tokyo 103-8433
TEL +81-3-3279-7417 FAX +81-3-3242-2270

[Out-of-hours points of contact]
Monitor supervisor: Hitoshi Kawamura (mobile phone: XXX-XXXX-XXXX)
Trial supervisor: Mitsumasa Nakamura (mobile phone: XXX-XXXX-XXXX)

8 Efficacy evaluation

8.1 Characteristics of demographic and other standard values

8.1.1 Items

Age, gender, medical history, corneal endothelial cell density and corneal thickness at the start of the administration period, and the presence or absence of morphological abnormalities in corneal endothelial cells

8.1.2 Calculations and statistics

In all the subjects, basic statistics are calculated for continuous data, and frequencies are indicated for binary or categorical data.

8.2 Identification of efficacy evaluation indices

In this clinical trial, the efficacy of the investigational drug is not evaluated.

9 Safety evaluation

9.1 Identification of safety evaluation indices

[Evaluation item 1]

Adverse events and adverse drug reactions

[Evaluation item 2]

Morphology of corneal endothelial cells

[Evaluation item 3]

Corneal endothelial cell density, corneal thickness, corneal volume, slit-lamp microscopy, visual acuity, and intraocular pressure

9.2 Evaluation and analysis

The names of adverse events are appropriately selected based on the description in the case report forms according to the "ICH International Pharmaceutical Glossary (Japanese version) (MedDRA/J^{TM})." For the selection, see "MedDRA Term Selection Considerations" and adverse event information. To examine medical appropriateness, seek advice from a medical professional as needed.

9.2.1 Analysis of each endpoint

[Endpoint for evaluation item 1]

Adverse events and adverse drug reactions

[Analytical methods]

Adverse events are summarized by PT (incidence, severity, and relevance), organ [System Organ Class (SOC)]. Adverse drug reactions are similarly summarized.

[Endpoint for evaluation item 2]

Morphological abnormality of corneal endothelial cells

[Analytical methods]

Intraindividual variation is examined.

[Endpoints of evaluation item 3]

Endpoint A: Amounts of change at each time point relative to those before the start of the administration period

Endpoint B: Difference in endpoint A between the right and left eyes of the subjects [Difference between K-115 ophthalmic solution (0.4%) administration (right eye) and non-administration (left eye)]

[Analytical methods]

Intraindividual variation is examined.

9.3 Additional analysis

Additional analysis is conducted as needed. Endpoints and analytical methods are described in a statistical analysis plan.

10 Statistical analysis

A statistical analysis plan, including detailed analytical methods, is created based on this chapter to conduct statistical analysis. The plan is created before fixing data, and the analysis is conducted after fixing data. Analytical items and time points may be selected based on the data.

10.1 Techniques of statistical analysis

Statistical analysis procedures, not specifically described, are conducted according to the SAS standard procedures (analysis version).

10.2 Target number of subjects and its rationale

[Target number of subjects]

6 (the number of subjects at the start of the administration period)

[Rationale]

This was determined in view of feasibility.

10.3 Level of significance and confidence coefficient

The level of significance is 5% on two-tailed tests.

The confidence coefficient is 95% on two-tailed tests.

10.4 Termination criteria

Statistical criteria for discontinuation are not determined.

10.5 Analysis sets

10.5.1 Efficacy analysis sets

Efficacy is not analyzed in this clinical trial.

10.5.2 Safety analysis set

Subjects with safety data at the start of the administration period and later, who receive K-115 ophthalmic solution (0.4%) at least once in the administration period, are included.

10.6 Handling of the subjects and data

After the end of the clinical trial, the sponsor fixes the data described in the case report form, and seeks advice from medical experts as needed to determine methods to handle the subjects and data.

10.6.1 Subject handling

The subjects not treated or completed as specified in the protocol are handled as shown in Table 10-1.

Table 10-1 Criteria for handling of subjects

Item	Conditions	Safety
1:Ineligible cases	Those not fulfilling the selection criteria or infringing on exclusion criteria	○
2:Terminated cases	Drop-outs in the administration period	○
3:Treatment violation cases	Not receiving a K-115 ophthalmic solution (0.4%) in the administration period	×
	No data after the start of the administration period (no visit after the start of the administration period)	×
	Poor compliance (<75%) with K-115 ophthalmic solution (0.4%) instillation between the start and end of the administration period.	○
	Those in whom the methods or time of implementation of observation/examinations partly disagree with the protocol	○
4:GCP non-conformity cases	Subjects such as those who have not been evaluated by the IRB, non-texecution of the trial contract, and not submitted consent	×

○: Adopted, ×: Not adopted

10.6.2 Data handling

[Prohibited concomitant treatments]

For subjects who violated the provisions of "7.2.1 Prohibited concomitant treatments," their data on safety evaluation indices in the evaluation period and later are individually considered.

[Instructions to subjects]

For subjects who violated the provisions of "7.3.2 Instructions to subjects" and these cases may affect safety evaluation, their data on safety evaluation indices in the evaluation period and later are individually considered.

[Observation/examinations, time of evaluation]

Data obtained at each implementing period specified in Table 7-2, "7.5.3 Ophthalmic inspection," and "7.5.4 Other inspection (optional inspection)" are used.

Multiple data within the acceptable range and data deviating from the acceptable range are individually considered.

[Modifications of inspection methods]

Modifications of ophthalmologic and other inspection methods are individually considered if any.

[Reference values]

Reference values are handled as missing values.

[Missing values]

If no data are available for any item specified in the protocol, it is handled as a missing value. Statistical models and imputation methods are not used for missing values.

[Time of instillation of the day]

Differences in instillation time on the night before visit and in the morning on the day of visit are individually considered.

[Poor instillation]

For subjects with poor compliance (<75%) with investigational drug instillation, data on the safety indices in the relevant periods are individually considered.

[Others]

Only data specified in the protocol are used.

10.7 Changes in the statistical analysis plan

In the preparation of the statistical analysis plan, if there are changes in the analytical methods from those prescribed in the protocol, the circumstances of the changes (time and reasons) must be recorded in the statistical analysis plan and clinical trial report.
